# Severe radiation-induced lymphopenia during postoperative radiotherapy or chemoradiotherapy has poor prognosis in patients with stage IIB-III after radical esophagectomy: A *post hoc* analysis of a randomized controlled trial

**DOI:** 10.3389/fonc.2022.936684

**Published:** 2022-09-08

**Authors:** Wenjie Ni, Zefen Xiao, Zongmei Zhou, Dongfu Chen, Qinfu Feng, Jun Liang, Jima Lv

**Affiliations:** ^1^ Department of Radiation Oncology, National Cancer Center/National Clinical Research Center for Cancer/Cancer Hospital, Chinese Academy of Medical Sciences and Peking Union Medical College, Beijing, China; ^2^ Department of Radiation Oncology, Beijing Shijitan Hospital, Capital Medical University, Beijing, China

**Keywords:** esophageal cancer, postoperative radiotherapy, lymphopenia, thoracic marrow, survival

## Abstract

**Objective:**

To investigate whether radiation-induced lymphopenia (RIL) affects survival and identify the predictors of RIL in postoperative esophageal cancer.

**Materials and methods:**

*Post hoc* analysis was conducted on data from 116 patients with esophageal cancer from a randomized controlled trial comparing adjuvant therapy with surgery alone. Doses of 54 Gy in 27 fractions was delivered in the postoperative radiotherapy (PORT) group and 50.4 Gy in 28 fractions combined with chemotherapy was delivered in postoperative concurrent chemoradiotherapy (POCRT) group. Blood counts were obtained before, during, and at first follow-up after treatment. Lymphopenia was graded per version 4.03 of the Common Terminology Criteria for Adverse Events. Disease-free survival (DFS) and overall survival (OS) were analyzed using the Kaplan-Meier method, and compared between groups using the log-rank test. Receiver operating characteristic curves identified thresholds for preventing grade 4 (G4) lymphopenia.

**Results:**

Median follow-up duration was 56.0 months. During treatment, 16 patients (13.8%) had G4 lymphopenia. All cases of G4 lymphopenia occurred in group PORT (30.2% vs 0.0%, p<0.001). Baseline absolute lymphocyte count was comparable between G1-3 and G4 patients (2.0 ± 0.8 *10^9^/L vs 1.7 ± 0.5 *10^9^/L; p=0.101). The 3-year DFS was significantly lower in group G4 lymphopenia than that in group G1-3 (31.3% vs 57.6%, p=0.036). The 3-year OS was comparable between both groups (50.0% vs 66.5%, p=0.095). Logistic regression analysis revealed that exposed more thoracic marrow (TM V20 ≥75%; TVB V20 ≥71%), heart (V15 ≥40%) and PTV (volume ≥507 ml) were associated with G4 lymphopenia (p<0.05).

**Conclusions:**

G4 RIL had poor disease-free survival, which may be related to more dose exposure of thoracic marrow and heart due to larger PTV. Reasonably reducing the radiation field combined with concurrent chemotherapy, or radiation dose constraints for these normal tissues may be sufficient to decrease the incidence of G4 lymphopenia, but further prospective trials are needed to verify the results.

**Clinical Trial Registration:**

clinicaltrials.gov, identifier NCT02279134

## Introduction

According to the Worldwide Esophageal Cancer Collaboration Investigators in 2016, about 58.7% of patients with esophageal cancer underwent surgical resection first (1). About 20% of patients with R0 resection in our hospital received postoperative radiotherapy. Especially for pathological stage III or lymph node positive esophageal cancer, it is reported that postoperative radiotherapy can significantly reduce the local regional recurrence rate and improve the survival rate (2–5). Furthermore, our research group has always devoted to the esophageal cancer clinical research after surgery alone, and has conducted many data analyses and improvement on the postoperative radiation field. Radiation therapy is an essential component of the treatment of esophageal cancer. However, it is reported that radiation may suppress host immunity, manifesting as lymphopenia (6, 7). Lymphocytes are extremely radiosensitive; therefore, relatively low doses can result in significant depletion of lymphocyte number (8). Radiation-induced lymphopenia (RIL) has been reported to adversely affect survival of patients with solid malignancies, such as glioma, lung cancer, and breast cancer (9–11). Severe lymphopenia during chemoradiotherapy is a strong predictor of poor outcomes and pathologic response rates in esophageal cancer (12–14). However, to the best of our knowledge, no study has been conducted on postoperative radiation therapy in esophageal cancer. In this study, we aimed to investigate whether RIL could affect survival, and identify the predictors of severe lymphopenia in postoperative esophageal cancer.

## Materials and methods

### Patients

This study was a *post hoc* analysis of data from a randomized controlled trial (NCT02279134) that was conducted from October 2014 through December 2019. The original trial recruited a total of 172 patients with esophageal cancer who had undergone radical esophagectomy. All patients were pathologically confirmed as stage IIB-III. The patients were randomly assigned to undergo surgery alone (SA group; n = 54), surgery and postoperative radiotherapy (PORT group; n = 54), or surgery and postoperative concurrent chemoradiotherapy (POCRT group; n = 64). The protocol has been described in detail elsewhere (15). Only patients who underwent PORT and POCRT were included in this study.

### Laboratory data

For the present study, the absolute lymphocyte count (ALC) of patients at different time points were collected from the case report forms. The ALC values at baseline (pre-ALC; within 1 week before radiation therapy), during radiation therapy (tested once a week), and within 3 months after treatment were available. Lymphopenia was graded according to version 4.03 of the Common Terminology Criteria for Adverse Events. The nadir ALC during the course of radiation therapy was classified as grade 0 (G0, ALC ≥ 1.0 × 10^9^/L), grade 1 (G1, 0.8 ≤ ALC < 1.0 × 10^9^/L), grade 2 (G2, 0.5≤ ALC < 0.8 × 10^9^/L), grade 3 (G3, 0.2 ≤ ALC < 0.5 × 10^9^/L), or grade 4 (G4, ALC < 0.2 × 10^9^/L).

### Dose-volume parameters

**Figure 1:**
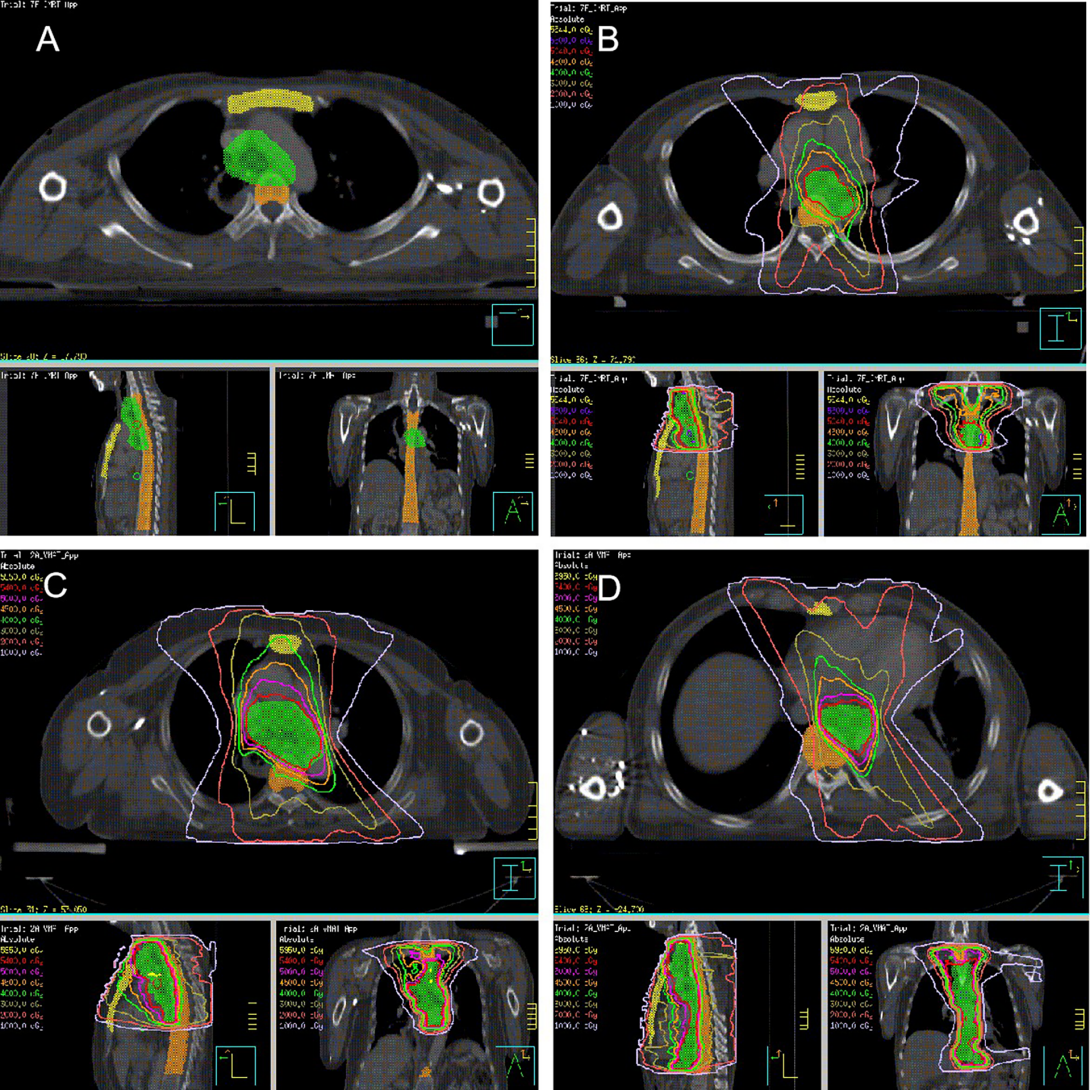
Radiation target (A. Thoracic marrow; yellow area, sternum; orange area, thoracic vertebral body; green area, PTV; **(B)** POCRT; **(C)** PORT, Upper-thoracic esophagus or Middle-thoracic esophagus with metastasis in 0 to 2 regional lymph nodes or metastasis in ≥ 3 regional lymph nodes in the mediastinum; **(D)** PORT, Lower-thoracic esophagus or middle-thoracic esophagus with metastasis in ≥ 3 regional lymph nodes distributed in two areas).

Thoracic marrow (TM), including sternum and thoracic vertebral body (TVB; the superior margin was 1.0 cm above the planning target volume (PTV) dose line and the inferior margin was the lower margin of T12 or PTV dose line), was contoured with the heart, lung, and spinal cord (**Figure 1A**). The relative volume of normal tissues at risk of receiving x Gy (V_x_) along with the mean dose (D_mean_) was calculated from the dose volume histogram.

### Treatment

#### Postoperative concurrent chemoradiotherapy

The borders of the clinical target volume (CTV) included the superior margin, which was the cricothyroid membrane for upper-thoracic tumors or the upper margin of the first thoracic vertebral body for middle-thoracic tumors. The inferior margin was 3.0 cm below the subcarina or the lower margin of the tumor bed (only for T4 lesions), including the lower cervical, bilateral supraclavicular region, and mediastinal stations 1R/L, 2R/L, 3p, 4R/L, 7, and part of 8 (**Figure 1B**). The prescription dose of PTV was 50.4 Gy (1.8 Gy/28 f). Paclitaxel (135-150 mg/m^2^) and cisplatin or nedaplatin (50-75 mg/m^2^) were administered concurrently. Chemotherapy was repeated every 28 days for two courses in the absence of disease progression or unacceptable toxicity.

#### Postoperative radiotherapy

The CTV was based on tumor and positive node location during surgery and pathological examination. The PTV was generated using a uniform 0.5 cm expansion around the CTV. Contouring of the CTV for tumors in different locations has been described in detail previously (15). **Figures 1C, D** illustrates the radiation target. The prescription dose was 54 Gy in 27 fractions of 2.0 Gy.

### Follow-up

After treatment, patients were followed up every 3 months for the first 2 years, every 6 months for the next 2 years, and once a year thereafter. Recurrence was confirmed using diagnostic imaging or histopathology.

Tumor recurrence in regional lymph nodes was defined based on the Union for International Cancer Control (7th edition) criteria. The regional lymph node groups included supraclavicular, mediastinal, and celiac area. Distant metastasis was defined as spread of tumor to distant organs or non-regional lymph nodes.

### Statistical analysis

Disease-free survival (DFS) was defined as the period from surgery to date of the first recurrence or death from any cause or censorship. Overall survival (OS) was defined as the interval from surgery to death from any cause or censorship. The Kaplan–Meier method was used to calculate DFS and OS, and the log-rank was used to determine the significance of differences. Logistic regression analysis was used to identify the factors of grade 4 lymphopenia. Receiver operating characteristic (ROC) curves identified thresholds to preventing G4 lymphopenia. All statistical analyses were performed using SPSS 20.0 (IBM Corp., Armonk, NY, USA). Two-tailed *p*<0.05 denoted statistically significant difference.

## Results

### Patients characteristics

Two patients lacking complete blood count data were excluded. Therefore, a total of 116 patients were included in the analysis. **Table 1** shows the patients characteristics based on the treatment modality, 53 and 63 patients were assigned to the PORT and POCRT groups, respectively. The volume of PTV in the PORT and POCRT group were 582.5 ± 109.5 ml and 464.8 ± 97.9 ml, respectively (p < 0.001). **Table 2** shows the demographic, tumor, and treatment characteristics between lymphopenic grades. Most of patients were male (89.7%); the average age was 57.3 years; and about half (44.8%) had a Karnofsky performance score of ≥90. Majority of patients (79.3%) had stage III disease. More patients underwent intensity-modulated radiotherapy (63.8%) rather than volumetric modulated arc therapy (36.2%). The ALC before treatment was comparable between patients in the G1-3 and G4 groups (2.0 ± 0.8 *10^9^/L vs 1.7 ± 0.5 *10^9^/L; p=0.101). All patients underwent different degrees of lymphopenia during treatment: G1 in 3 (2.6%) patients, G2 in 22 (19.0%) patients, G3 in 75 (64.7%) patients, and G4 in 16 (13.8%) patients. Patients with G4 lymphopenia only underwent PORT. The volume and mean dose of PTV were higher in group G4 (p < 0.05). All other characteristics were well balanced between the two groups.

**Table 1:** Comparison of patient characteristics between PORT and POCRT.

	Frequency, n (%)	PORT, n (%)	POCRT, n (%)	P
Gender Male Female	104 (89.7) 12 (10.3)	48 (90.6) 5 (9.4)	56 (88.9) 7 (11.1)	1.000
Age (mean ± SD, years)	57.3 ± 6.3	57.9 ± 6.9	56.7 ± 5.7	0.326
Kps 80 90 100	64 (55.2) 50 (43.1) 2 (1.7)	34 (64.2) 18 (34.0) 1 (1.9)	30 (47.6) 32 (50.8) 1 (1.6)	0.136
Tumor location Upper Middle Lower	6 (5.2) 49 (42.2) 61 (52.6)	3(5.7) 21(39.6) 29 (54.7)	3 (4.8) 28 (44.4) 32 (50.8)	0.906
TNM stage (UICC 7th) IIB IIIA IIIB IIIC	24 (20.7) 47 (40.5) 30 (25.9) 15 (12.9)	13 (24.5) 17 (32.1) 17 (32.1) 6 (11.3)	11 (17.5) 30 (47.6) 13 (20.6) 9 (14.3)	0.262
Differentiation degree Well Moderate Poor	9 (7.8) 61 (52.6) 46 (39.7)	6 (11.3) 24(45.3) 23(43.4)	3(4.8) 37(58.7) 23(36.5)	0.221
Radiation modality IMRT VMAT	74 (63.8) 42 (36.2)	32 (60.4) 21 (39.6)	42 (66.7) 21 (33.3)	0.562
PTV volume (mean ± SD, ml)	519.5 ± 118.7	582.5 ± 109.5	464.8 ± 97.9	<0.001
Lymphopenia G1-3 G4	100 (86.2) 16 (13.8)	37 (69.8) 16 (30.2)	63 (100) 0 (0)	<0.001

PORT, postoperative radiotherapy; POCRT, postoperative concurrent chemoradiotherapy; G, grade; SD, standard deviation; Kps, Karnofsky performance score; UICC, Union for International Cancer Control; IMRT, intensity-modulated radiotherapy; VAMT, volumetric modulated arc therapy; PTV, planning target volume.

**Table 2:** Comparison of patient characteristics between lymphopenic grades.

	Frequency, n (%)	G1–3, n (%)	G4, n (%)	P
Gender Male Female	104 (89.7) 12 (10.3)	90 (90.0) 10 (10.0)	14 (87.5) 2(12.5)	0.671
Age (mean ± SD, years)	57.3 ± 6.3	57.1 ± 6.2	58.1 ± 6.8	0.555
Kps 80 90 100	64 (55.2) 50 (43.1) 2 (1.7)	52 (52.0) 46(46.0) 2(2.0)	12 (75.0) 4 (25.0) 0 (0.0)	0.241
Tumor location Upper Middle Lower	6 (5.2) 49 (42.2) 61 (52.6)	5 (5.0) 42 (42.0) 53 (53.0)	1 (6.2) 7 (43.8) 8 (50.0)	0.963
TNM stage (UICC 7th) IIB IIIA IIIB IIIC	24 (20.7) 47 (40.5) 30 (25.9) 15 (12.9)	22 (22.0) 38 (38.0) 27 (27.0) 13 (13.0)	2(12.5) 9(56.2) 3 (18.8) 2 (12.5)	0.610
Differentiation degree Well Moderate Poor	9 (7.7) 61 (52.6) 46 (39.7)	6 (6.0) 56 (56.0) 38 (38.0)	3 (18.8) 5 (31.2) 8 (50.0)	0.082
Radiation modality IMRT VMAT	74 (63.8) 42 (36.2)	62 (62.0) 38 (38.0)	12(75.0) 4 (25.0)	0.315
PTV volume (mean ± SD, ml)	519.5 ± 118.7	507.3 ± 119.8	594.6 ± 79.9	0.006
PTV mean dose (mean ± SD, Gy)	55.0 ± 2.1	54.7 ± 2.1	56.6 ± 1.7	0.001
Concurrent chemotherapy Yes No	63 (54.3) 53 (45.7)	63 (63.0) 37 (37.0)	0 (0.0) 16 (100.0)	<0.001
Pre-ALC (mean ± SD, *10^9^/L)	2.0 ± 0.8	2.0 ± 0.8	1.7 ± 0.5	0.101

G, grade; SD, standard deviation; Kps, Karnofsky performance score; UICC, Union for International Cancer Control; IMRT, intensity-modulated radiotherapy; VAMT, volumetric modulated arc therapy; PTV, planning target volume; ALC, absolute lymphocyte count.

### Correlation between lymphopenia and survival

**Figure 2:**
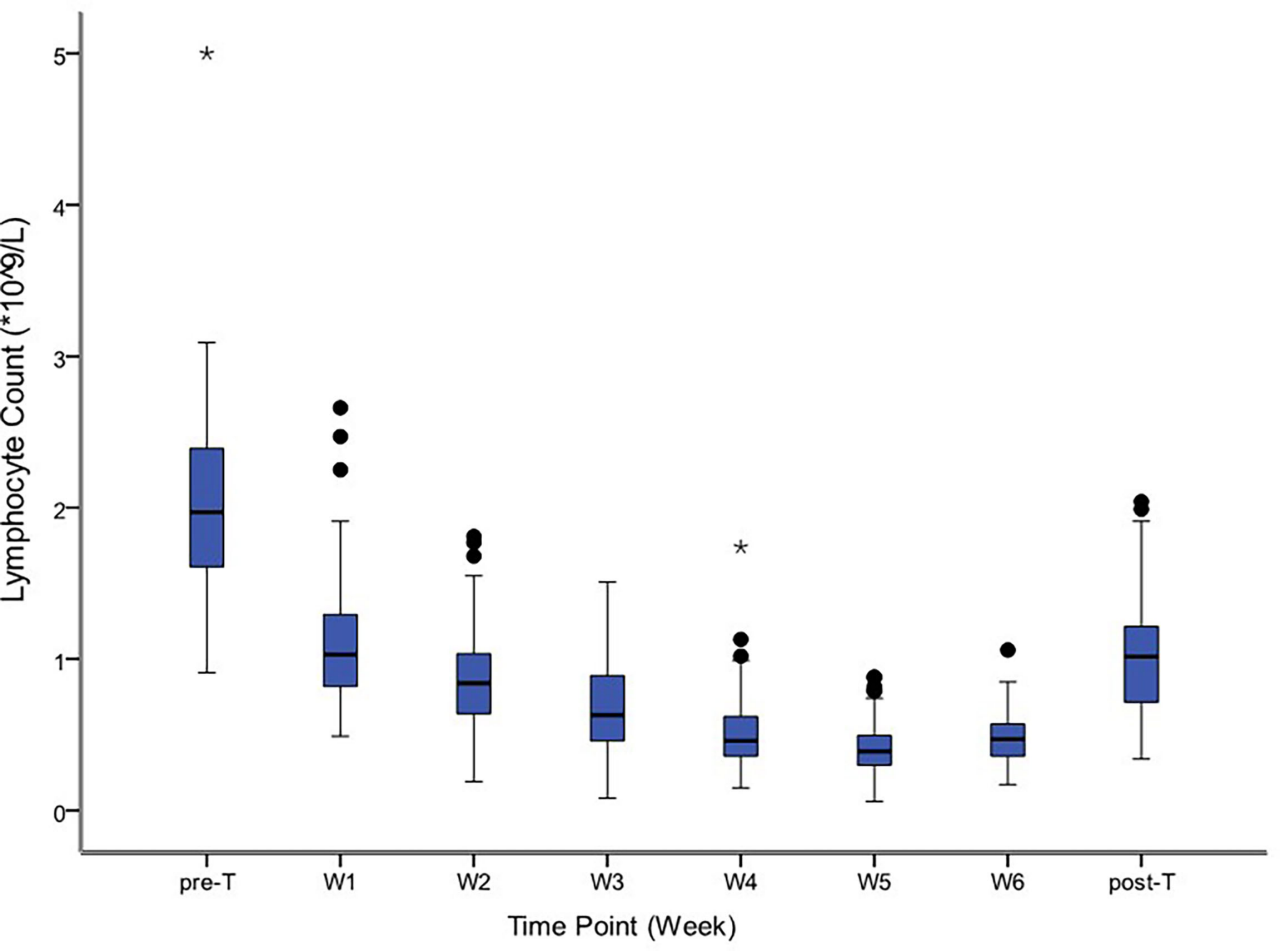
Distribution of absolute lymphocyte counts before, during and after treatment. The symbol * means outlier.

**Figure 3:**
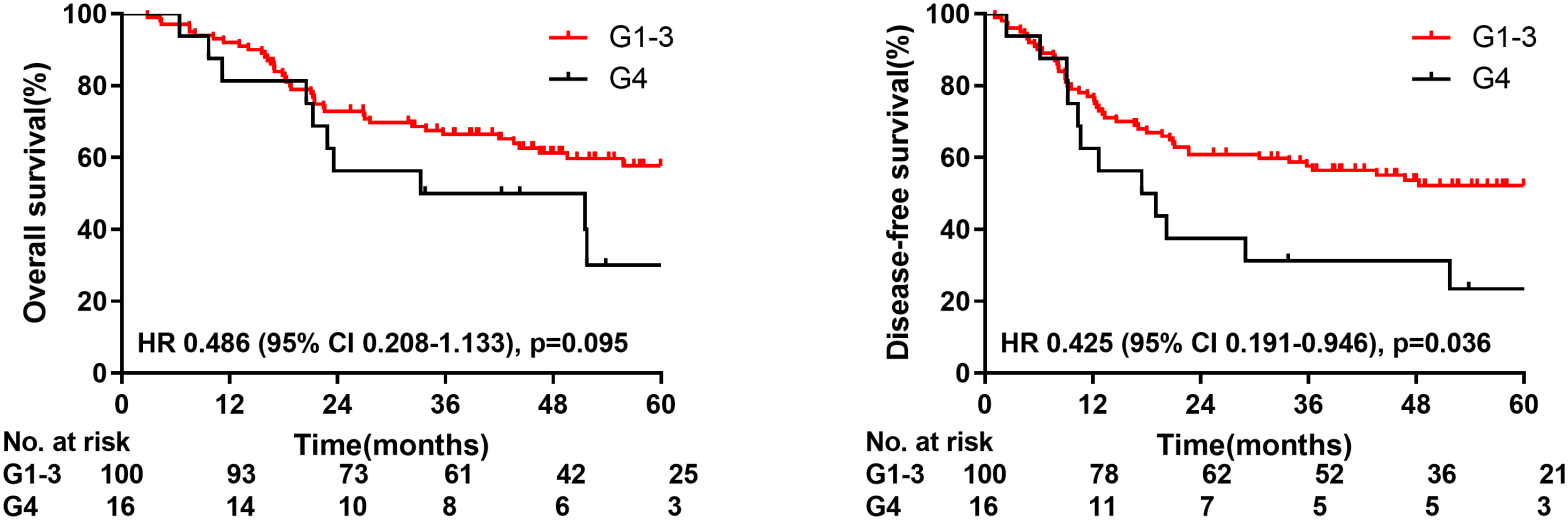
Overall survival and disease-free survival for patients with radiation induced lymphopenia.

The median time of radiation therapy was 5.4 weeks. The ALC decreased gradually during treatment, and reached the nadir in the fifth week (**Figure 2**). The last follow-up date was January 25, 2021; the median follow-up period was 56.0 months. The median OS time was 33.2 months in group G4; however, the OS in group G1-3 was not reached. The 1-year, 3-year, and 5-year OS were 81.3%, 50.0%, and 30.0%, respectively, in the G4 group, compared with 92.0%, 66.5%, and 57.7%, respectively, in the G1-3 group (HR: 0.486, 95% CI: 0.208-1.133, p=0.095). The median DFS time was 17.4 months in group G4, but not attained in group G1-3. The 1-year, 3-year, and 5-year DFS were 62.5%, 31.3%, and 23.4%, respectively, in the G4 group, compared with 77.0%, 57.6%, and 52.2%, respectively, in the G1-3 group (HR: 0.425, 95% CI: 0.191-0.946, p=0.036) (**Figure 3**).

### Predictors of lymphopenia

**Table 3** shows the relationship between lymphopenia during treatment and different clinical characteristics. Patients age, gender, and radiation technique were not significantly associated with the risk of G4 lymphopenia. In terms of dosimetric predictors, the radiation dose of TM, TVB, Heart, PTV, and PTV volume were all associated with higher rates of G4 lymphopenia (all p<0.05). Sternum D_mean_, V10, and V20 were predictors of G4 lymphopenia (p<0.05).

**Table 3:** Logistic regression analysis of factors associated with grade 4 lymphopenia.

	OR	95% CI	P
Age	1.026	0.942–1.118	0.552
Male vs. Female	1.286	0.255–6.492	0.761
TM Dmean	1.176	1.084–1.275	<0.001
TVB Dmean	1.159	1.078–1.246	<0.001
Sternum Dmean	1.109	1.001–1.228	0.048
TM V5	1.091	1.040–1.144	<0.001
TVB V5	1.075	1.034–1.118	<0.001
Sternum V5	2.421	0.576–10.189	0.228
TM V10	1.087	1.039–1.137	<0.001
TVB V10	1.073	1.034–1.114	<0.001
Sternum V10	1.225	1.040–1.443	0.015
TM V20	1.083	1.040–1.127	<0.001
TVB V20	1.074	1.036–1.113	<0.001
Sternum V20	1.045	1.004–1.088	0.032
TM V30	1.063	1.030–1.097	<0.001
TVB V30	1.061	1.031–1.091	<0.001
Sternum V30	0.979	0.940–1.020	0.307
TM V40	1.057	1.019–1.096	0.003
TVB V40	1.057	1.023–1.092	0.001
Sternum V40	0.995	0.957–1.036	0.815
TM V50	1.100	1.018–1.189	0.016
TVB V50	1.091	1.018–1.170	0.014
Sternum V50	1.037	0.959–1.123	0.363
Heart Dmean	1.152	1.062–1.251	0.001
Heart V15	1.056	1.024–1.090	0.001
Heart V20	1.050	1.023–1.078	<0.001
Heart V30	1.083	1.036–1.133	<0.001
Heart V40	1.122	1.049–1.201	0.001
Heart V50	1.269	1.112–1.449	<0.001
PTV Volume	1.006	1.002–1.011	0.009
PTV Dmean	1.566	1.170–2.095	0.003
IMRT vs. VMAT	0.544	0.164–1.808	0.320

OR, odd ratio; CI, confidence interval; TM, thoracic marrow; TVB, thoracic vertebral body; PTV, planning target volume; Dmean, mean dose; Vx, relative volume of receiving x Gy.

We further explored the optimal cut-off points of the dosimetric variables significantly associated with G4 lymphopenia (TM, TVB, Heart, PTV Volume, PTV D_mean_) using ROC curve analysis (**Table 4**). The ROC curves for partial important variables were available on line (**Supplementary Figures 1–4**).

**Table 4:** ROC curve cut-off points for prevention of grade 4 lymphopenia.

	Cut-off point	AUC	P
TM Dmean	< 32Gy	0.837	< 0.001
TM V5	< 79%	0.853	< 0.001
TM V10	< 78%	0.847	< 0.001
TM V20	< 75%	0.860	< 0.001
TM V30	< 60%	0.813	< 0.001
TM V40	< 35%	0.758	0.001
TM V50	< 11%	0.698	0.011
TVB Dmean	< 32Gy	0.850	< 0.001
TVB V5	< 74%	0.863	< 0.001
TVB V10	< 74%	0.871	< 0.001
TVB V20	< 71%	0.892	< 0.001
TVB V30	< 62%	0.854	< 0.001
TVB V40	< 35%	0.788	< 0.001
TVB V50	< 17%	0.700	0.010
Heart Dmean	< 14Gy	0.809	< 0.001
Heart V15	< 40%	0.847	< 0.001
Heart V20	< 48%	0.823	< 0.001
Heart V30	< 23%	0.795	< 0.001
Heart V40	< 10%	0.781	< 0.001
Heart V50	< 2%	0.818	< 0.001
PTV Volume	< 507ml	0.742	0.002
PTV Dmean	< 55Gy	0.749	0.001

ROC, receiver operating characteristic; AUC, area under curve; TM, thoracic marrow; TVB, thoracic vertebral body; PTV, planning target volume; Dmean, mean dose; Vx, relative volume of receiving x Gy.

## Discussion

As we all know, the lymph node metastasis of esophageal cancer occurs early and widely, and the recurrence of lymph nodes after radical resection is the main reason, accounting for 23.8%-58% (16), especially for patients with pathological positive lymph nodes. Therefore, how to balance the effective radiation field is the focus of our research. This *post hoc* analysis is from a prospective randomized controlled trial after the third modified radiation field, which showed that postoperative adjuvant therapy could improve the survival rate compared with surgery alone.

Our study revealed that DFS was worse in patients with G4 lymphopenia during PORT for esophageal cancer. The predictors of G4 RIL include the radiation volume of PTV and the adjacent hematopoietic system, such as sternum, thoracic vertebral body and heart, however it seems to have little relationship with chemotherapy. Lymphopenia is known to be one of the manifestations of immunosuppression. Many clinical studies have shown that it is a predictor of poor prognosis in pancreatic cancer, brain tumor, non-small cell lung cancer, and nasopharyngeal carcinoma (17–20). According to recent studies, patients with lymphopenia during radical or neoadjuvant chemoradiotherapy of esophageal cancer have a poor prognosis and low complete pathologic response rate (12–14). Our study showed that lymphocytes were extremely sensitive to radiation. Lymphocytes decreased at the beginning of radiotherapy and sharply with the accumulation of radiation dose. Radiation doses, as low as 2 Gy, can inactivate about 50% of circulating lymphocytes in the radiation field, resulting in RIL during radiotherapy (21, 22). In a malignant glioma model, 60 Gy prescription dose irradiates the brain at a dose of 2 Gy per fraction, resulting in an average dose of 2 Gy for circulating lymphocytes, and almost all circulating blood is at least irradiated 0.5 Gy (8). T lymphocytes are an important part of cellular immunity. Cytotoxic CD8+ T lymphocytes act as effector cells; they directly kill abnormal cells and secrete proinflammatory cytokines (23). Therefore, radiation-induced reduction of CD8+ T lymphocytes may have a negative effect on cell-mediated immunity, because even if the number of lymphocytes recovers after radiotherapy, the newly produced immature T lymphocytes cannot produce antitumor effects. Regulatory T cells (Tregs), another T cell subtype, are known to be involved in immunosuppression (24). Muroyama et al. (25) found that the phenotypic and functional inhibitory Treg cells number increases in a tumor microenvironment after irradiation of tumor with 10 Gy in mice. According to Oweida et al. (26, 27), the combination of radiotherapy and immunotherapy with Treg targeted inhibitors can inhibit tumor growth. Since Treg is relatively resistant to radiation, surviving Treg cells are usually assumed to have the ability to inhibit the recovery of effector T cells during lymphocyte recovery (28). Clinical study findings also showed that a high proportion of CD8+ T/Treg cells predicted a better therapeutic response (29). Therefore, the effect of lymphopenia on the survival of patients could be mainly due to the extensive effect of radiotherapy on the number and function of effector T lymphocytes in blood circulation. Moreover, Treg cells are radiation-resistant and affect the recovery of effector T lymphocytes after radiotherapy, resulting in the decline of cellular immune function, early recurrence, and worse prognosis.

Lymphopenia is mainly due to the reduction in number of mature lymphocytes in peripheral blood and the production of lymphocytes in hematopoietic organs after radiation. The heart is highly vascularized. The thoracic marrow is the main hematopoietic organ of adults. The heart and sternum are located in front of the esophagus and the thoracic vertebra is located behind the esophagus. In this study, we found that the irradiated volume and dose of thoracic vertebra, heart, and PTV during postoperative radiotherapy of esophageal cancer were the main factors causing G4 lymphopenia. Fang (13, 30) and van Rossum (31) reported that G4 lymphocytes decreased more significantly in patients with larger PTV in radical chemoradiotherapy of esophageal cancer, which is consistent with the results of our study. Davuluri (12) reported that the incidence of G4 lymphopenia in patients with lesions in the lower sections of the esophagus is higher than that in patients with lesions in the middle and upper sections of the esophagus. Considering that the lesions in the lower sections of the esophagus are adjacent to the heart and spleen, which are rich in blood, a large number of lymphocytes are irradiated. Saito (32) previously reported that the average irradiation dose of spleen in chemoradiotherapy of esophageal cancer can predict G4 lymphopenia. Besides, the exposure of thoracic vertebra in esophageal cancer radiotherapy has been reported to be related to more grade 3 hematological toxicity (33–35). According to Newman (36), lymphopenia during chemoradiotherapy of esophageal cancer is closely related to the volume of irradiated thoracic vertebral body, which is consistent with our findings.

Proton radiotherapy in malignant tumors has been more widely used than photon radiotherapy for its physical advantages. Mohan (37) reported that proton radiotherapy could better reduce the incidence of G3 lymphopenia in glioblastoma than photon radiotherapy. Nichols (38) also revealed that proton radiotherapy could better reduce the mean radiation dose of the lungs by 33% and bone marrow V10 by 30% than photon radiotherapy. Shiraishi (39) and Liu (40) reported that proton radiation to the heart has lower doses than photon radiation. Several studies have reported that proton radiotherapy has a lower incidence of G4 lymphopenia than photon radiotherapy during chemoradiotherapy in esophageal cancer (30, 41, 42). Our study reveals that a greater volume and dose of PTV has a higher irradiation dose of thoracic marrow and heart, which results in a more obvious decrease in the number of peripheral blood lymphocytes.

Our study reported the cut-off values of PTV, heart, and thoracic marrow necessary to prevent the incidence of G4 lymphopenia. According to the prospective randomized controlled trial by Ni et al. (43), for patients with pathological stage IIB–III esophageal squamous cell carcinoma after radical surgery, POCRT, which reduced the radiation field to 3 cm below the carina and reduced the radiation dose to 50.4 Gy, did not increase the in- or out-of-field recurrence. Additionally, the survival rate was more comparable than with the PORT. POCRT appears to be an effective and safe treatment. Based on findings from the previous and present trials, POCRT can be considered for these patients to ensure a smaller PTV volume and dose to reduce the exposure of the heart and thoracic marrow and prevent severe lymphopenia. In the event where POCRT cannot be performed, attention should be paid to the protection of the heart and thoracic marrow, and corresponding radiation dose constraints should be given to prevent lymphopenia. Therefore, under the condition of reasonably reducing the postoperative irradiation field, synchronous chemotherapy should be strengthened to reduce the impact on lymphocytes and reduce the impact on survival. Of course, the postoperative irradiation field should be designed according to the recurrence sites and rates after esophagectomy, and the irradiation dose of normal tissue should be considered at the same time, so as to reduce the recurrence rate and convert it into the benefit of survival without increasing toxic and side effects.

In addition, actively search for drugs to enhance immunity or promote lymphocyte recovery is the direction of future research. Zheng (44) found that after a single low-dose whole-body irradiation in the mouse lung melanoma model, cinnamon effectively improved the imbalance of T cell subsets and promoted effective antitumor immunity by promoting the proliferation of Th1 and inhibiting the expansion of Th17 and Treg cells. In addition, an experimental study has also shown that exogenous IL-7 delivered to the irradiated animal model can not only restore the lymphocyte count but also enhance the antitumor effect. Exogenous IL-7 is helpful to overcome RIL and improve the therapeutic effect combined with radiotherapy (45). However, these findings need to be verified by future clinical studies.

The limitation of this study is that the sample size is relatively small. We expect to continue accumulating more cases and prolong the follow-up time.

## Conclusion

G4 lymphopenia had poor DFS, and the radiation volume of thoracic marrow, heart, and PTV may predict G4 lymphopenia in postoperative esophageal cancer. Radiation dose constraints for these normal tissues may be sufficient to decrease G4 lymphopenia, but further prospective trials are needed to verify the results.

## Data availability statement

The original contributions presented in the study are included in the article/**Supplementary Material**. Further inquiries can be directed to the corresponding author.

## Ethics statement

The studies involving human participants were reviewed and approved by Ethics Committee of Cancer Institute and Hospital, Chinese Academy of Medical Sciences, Beijing. The patients/participants provided their written informed consent to participate in this study.

## Author contributions

Conceptualization: ZX. Project planning: WN, ZX. Writing: WN. Statistical counseling: WN. Editing: ZX, ZZ, DC, QF, JL, JML. All authors provided review of the manuscript. All authors read and approved the final manuscript.

## Funding

This work were supported by the Capital Fund for Health Improvement and Research [grant number 2016-2-4021] and Youth Fund of Beijing Shijitan Hospital (grant number 2020-q12). The manuscript has been peer reviewed by the funding body. The funding source has no role in study design, data collection, analysis, interpretation, the writing of the manuscript, or the decision to submit the current study.

## Acknowledgments

We thank all participants of this trial, and all investigators who devote their time and passion in the implementation of this study.

## Conflict of interest

The authors declare that the research was conducted in the absence of any commercial or financial relationships that could be construed as a potential conflict of interest.

## Publisher’s note

All claims expressed in this article are solely those of the authors and do not necessarily represent those of their affiliated organizations, or those of the publisher, the editors and the reviewers. Any product that may be evaluated in this article, or claim that may be made by its manufacturer, is not guaranteed or endorsed by the publisher.
