# Supplementary material for: Severe radiation-induced lymphopenia during postoperative radiotherapy or chemoradiotherapy has poor prognosis in patients with stage IIB-III after radical esophagectomy: A post hoc analysis of a randomized controlled trial
Source: Front Oncol. 2022 Sep 8;12:936684. doi: 10.3389/fonc.2022.936684 (PMC9492938; doi:10.3389/fonc.2022.936684)
Supplement: Supplementary file 1 [file DataSheet_1.docx]

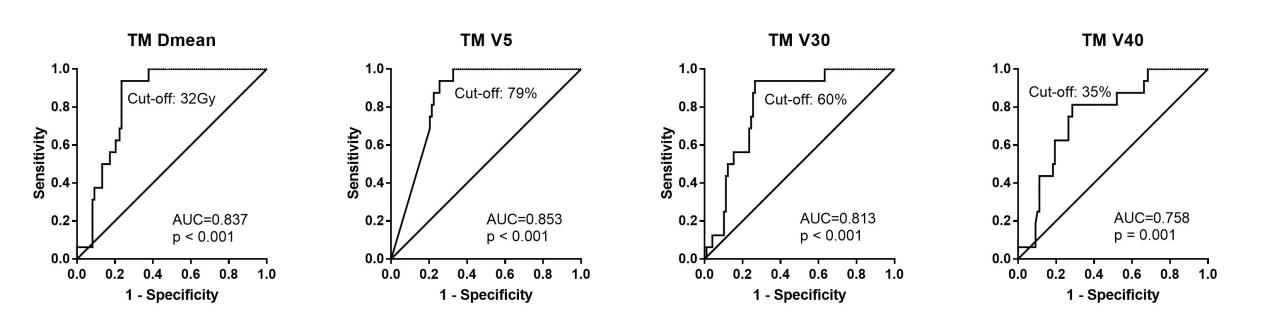


Supplement figure 1. The ROC curves for TM to avoid G4 lymphopenia


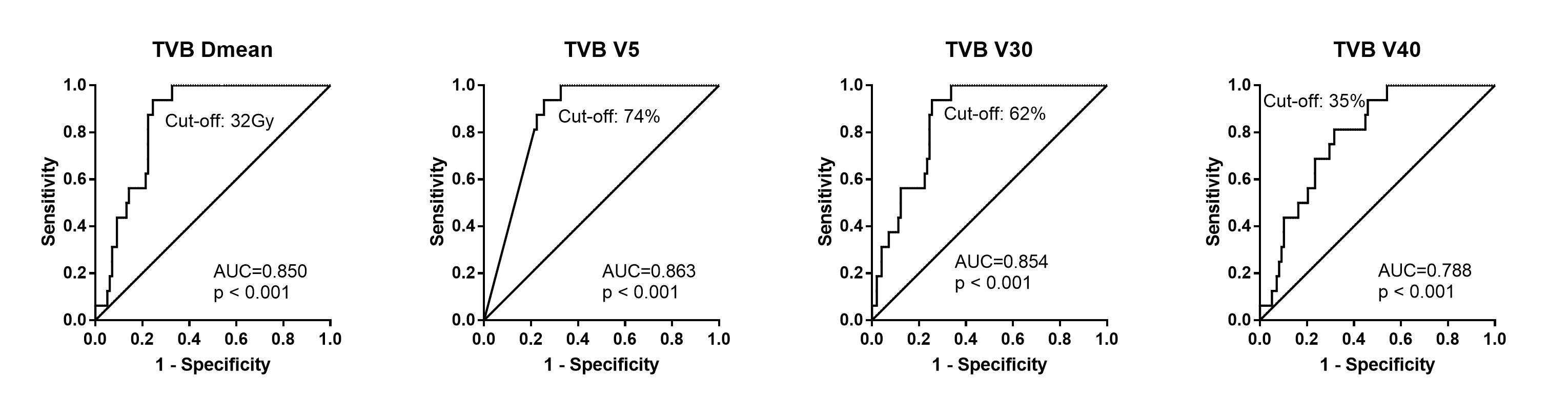


Supplement figure 2. The ROC curves for TVB to avoid G4 lymphopenia


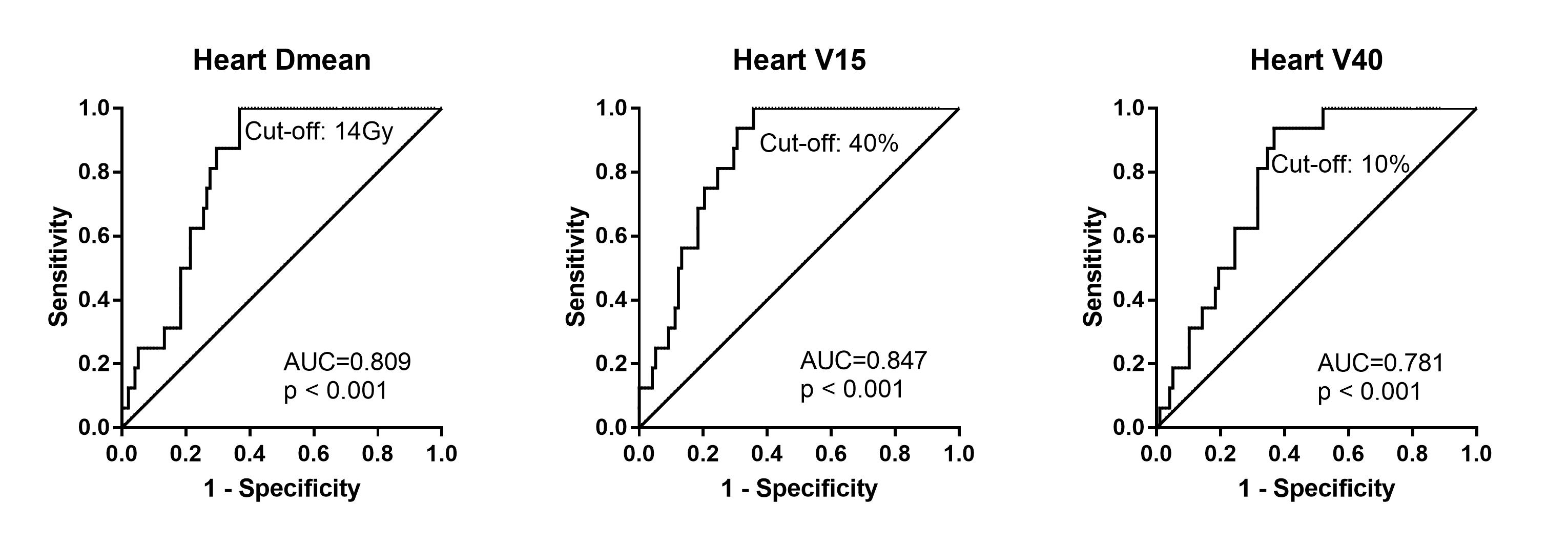


Supplement figure 3. The ROC curves for Heart to avoid G4 lymphopenia


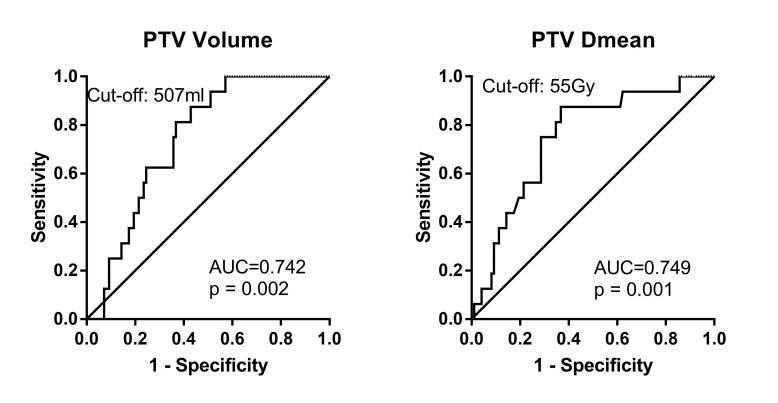


Supplement figure 4. The ROC curves for PTV to avoid G4 lymphopenia
